# Larynx Trauma and Hyoid Bone Fracture after Bite Injury in Dog: Case Report

**DOI:** 10.3389/fvets.2016.00064

**Published:** 2016-08-16

**Authors:** George Manchi, Mathias M. Brunnberg, Muhammad Shahid, Ahmad Al Aiyan, Leo Brunnberg, Silke Stein

**Affiliations:** ^1^Faculty of Veterinary Medicine, Small Animal Clinic, Freie University Berlin, Berlin, Germany; ^2^Veterinary Medicine Department, College of Food and Agriculture, United Arab Emirates University, Al Ain, United Arab Emirates

**Keywords:** larynx trauma, hyoid fracture, bite, canine, dyspnea, neck, shock

## Abstract

An 8-year-old male Jack Russell crossbreed dog was admitted to our hospital with dyspnea and shock following a dog-bite injury on the ventral neck. Radiographs revealed subcutaneous emphysema and bilateral thyrohyoid bone fractures. Intraoperatively, rupture of both sternohyoid muscles, both hyoepiglotticus muscles, both thyrohyoid muscles, and a partial cranial rupture of the superficial sphincter colli muscle were detected. Part of the epiglottis was detached from the thyroid cartilage. The patient’s severed muscles and torn epiglottis were reattached using a simple interrupted suture pattern. Hyoepiglotticus muscles could not be identified. The bilateral thyrohyoid bone fractures were repaired with intraosseous wire suture. A temporary tracheostomy tube and an esophageal feeding tube were placed postoperatively. The dog was discharged after 8 days, re-examined at 2 and 6 months and laryngeal and pharyngeal function were evaluated as normal. To the authors’ knowledge, this is the first report of a dog that presented with laryngeal trauma with hyoid bone fracture and acute dyspnea that underwent surgical treatment resulting in an acceptable outcome.

## Case Presentation

An 8-year-old male Jack Russell crossbreed dog weighing 10 kg was admitted to the emergency service at the small animal hospital of Freie Universität Berlin. The owner reported that a German shepherd bit his dog in the throat (pharyngeal/laryngeal) region approximately an hour before presentation. The dog was alert, restless, and was able to stand but could not walk. He was in sternal recumbency with his neck extended. Blood-stained saliva was dripping from his mouth. Subcutaneous emphysema was palpable on the ventral neck. The patient was assessed to be in shock upon presentation, and successful therapy was instituted preoperatively.

**Figure 1:**
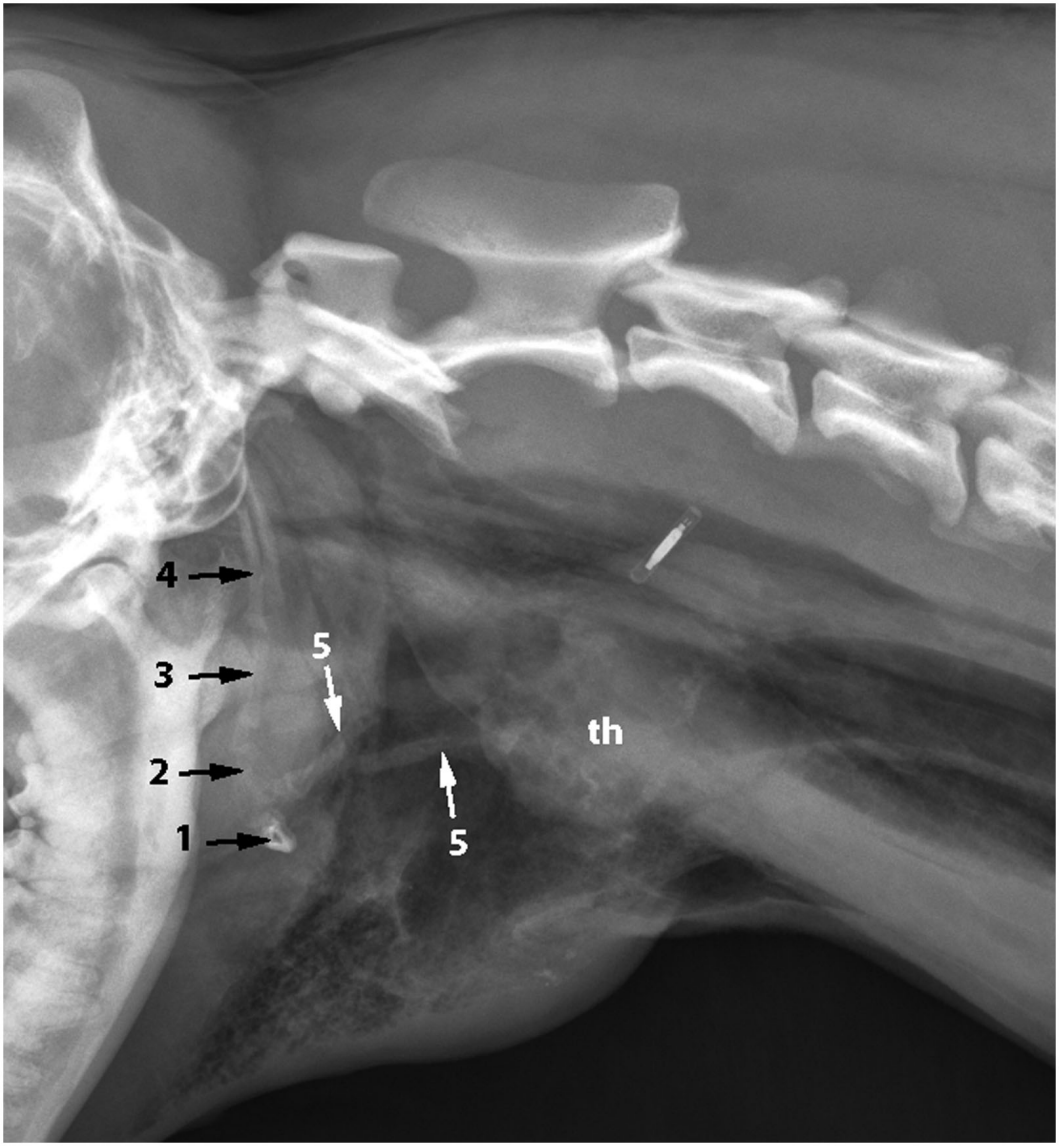
**Latero-lateral radiograph of the neck: bilateral fracture of the thyrohyoid bones (white arrows)**. The hyoid bones (1–5) and thyroid cartilage (th) can be identified.

**Figure 2:**
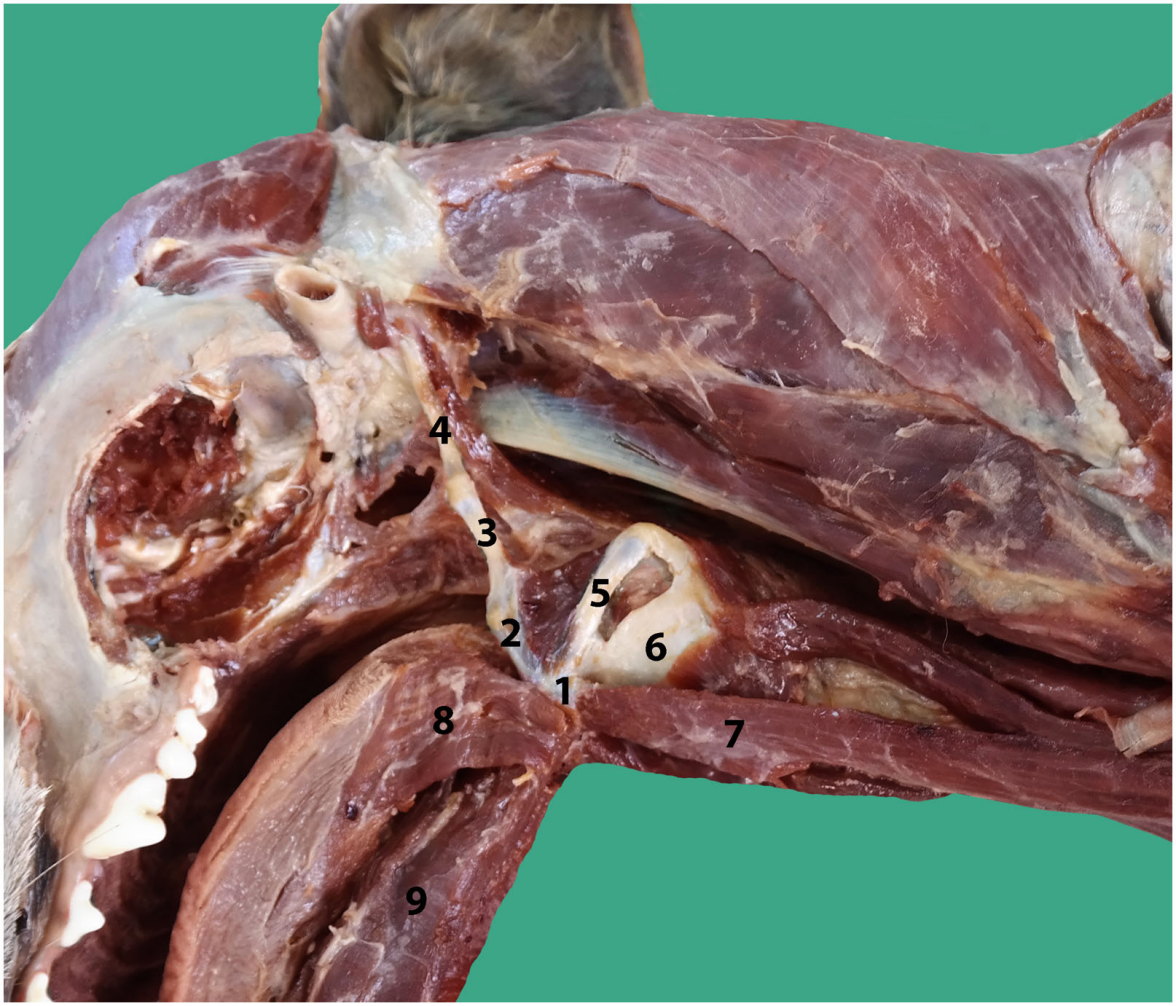
**Anatomy of the hyoid bone in a canine cadaver (1–5), lateral aspect: (1) Basihyoid, (2) Ceratohyoid, (3) Epihyoid, (4) Stylohyoid, (5) Thyrohyoid, (6) Thyroid cartilage, (7) Sternohyoid muscle, (8) Hyoglossal muscle, and (9) Geniohyoid muscle**.

Emergency treatment included oxygen supplementation with a nasal oxygen catheter. After cardiovascular stabilization, the dog was sedated with a minimal dose of acepromazine (0.02 mg/kg IV) and metamizole (30 mg/kg IV). Radiographs of the neck (latero-lateral and ventro-dorsal) and thorax (latero-lateral and dorso-ventral) were taken. They revealed subcutaneous air bubbles consistent with subcutaneous emphysema near the larynx and dorsal to the neck, a pneumomediastinum, and bilateral fractures of the thyrohyoid bones (Figures 1 and 2).

The dog was prepared for surgery with the owner’s consent; endoscopy and surgery were performed under general anesthesia. For premedication, the dog was administered diazepam (0.5 mg/kg IV) and levomethadone (0.4 mg/kg IV). Thorough examination of the larynx, oral cavity, and pharynx revealed sublingual hematoma and swelling around the base of the tongue. The epiglottis could not be identified because of a massive hematoma between the tongue and larynx. The mucosa of the arytenoid was moderately edematous.

Following laryngoscopy, anesthesia was induced with propofol (1 mg/kg IV), and the patient was intubated with a small endotracheal tube (5.5 mm) since intubation was difficult. An esophageal endoscopy with a 5.0 mm × 530 mm, 0°, rigid endoscope did not reveal any esophageal injuries. During a brief period of extubation, endoscopic examination of larynx and cervical trachea was performed, which showed no evidence of perforation or laceration; however, mucosal surfaces of the esophagus, larynx, and trachea were covered with blood. Anesthesia was maintained with isoflurane and oxygen, and the dog was ventilated throughout the surgical procedure. Monitoring was conducted using capnography, pulse oximetry, ECG, and oscillometric blood pressure measurement.

Prior to surgery, the ventral neck was clipped from the mandible to the manubrium, revealing severe bruising of the skin ventral to the larynx and the neck. The skin was not perforated. The patient was positioned in dorsal recumbency with the neck extended, and the surgical site was aseptically prepared.

**Figure 3:**
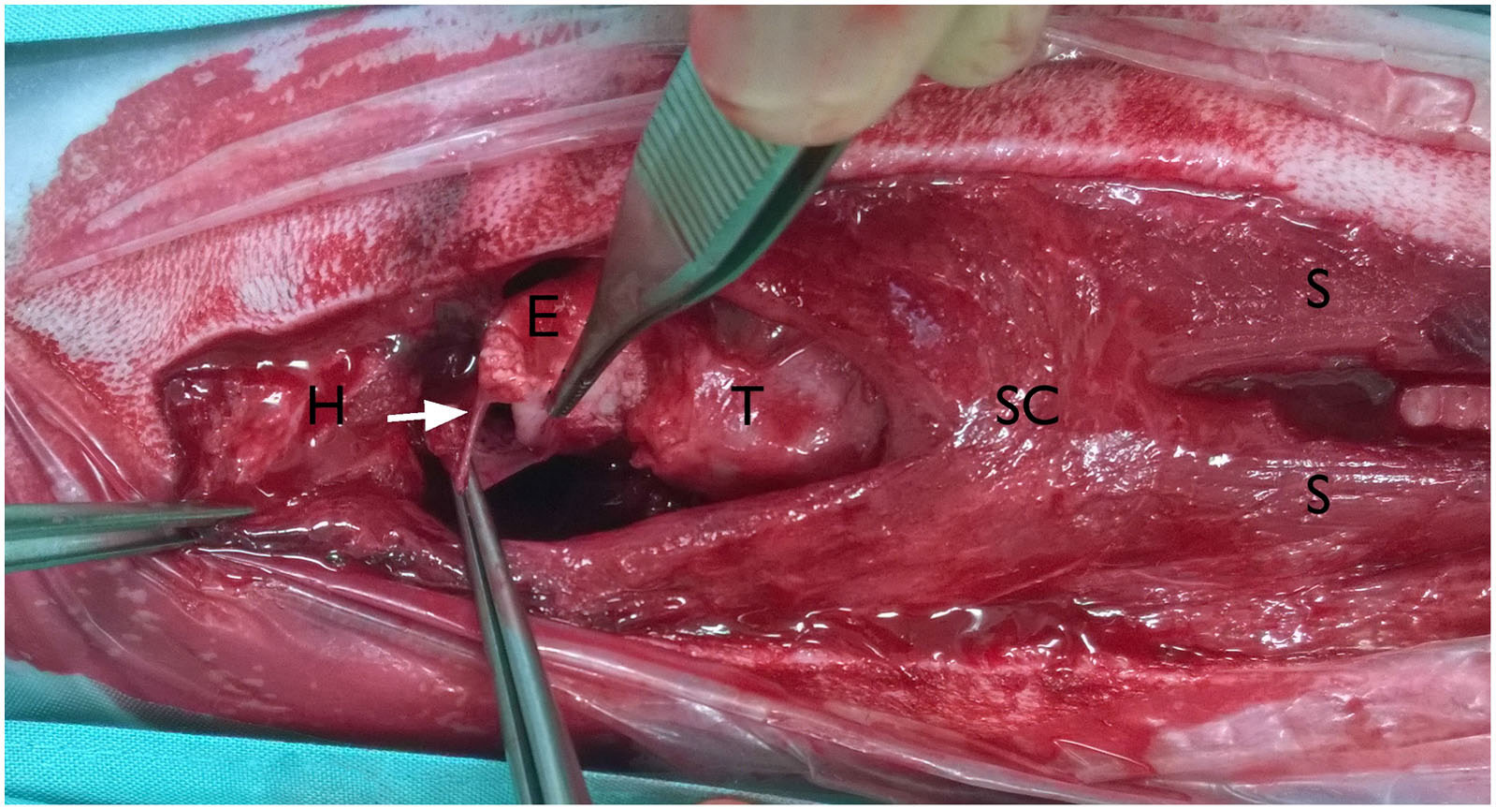
**Intraoperative pictures; ventral approach to the larynx: the head is on the left side of the picture**. Basihyoid bone (H) with part of the sternohyoid muscles displaced cranially, cranial rupture of both sternohyoid muscles (S) and separation from the basihyoid bone, tear in the mucosa ventral to the epiglottis, piriform recess is opened (arrow), superficial sphincter colli muscle (SC), epiglottis (E), and thyroid cartilage (T).

**Figure 4:**
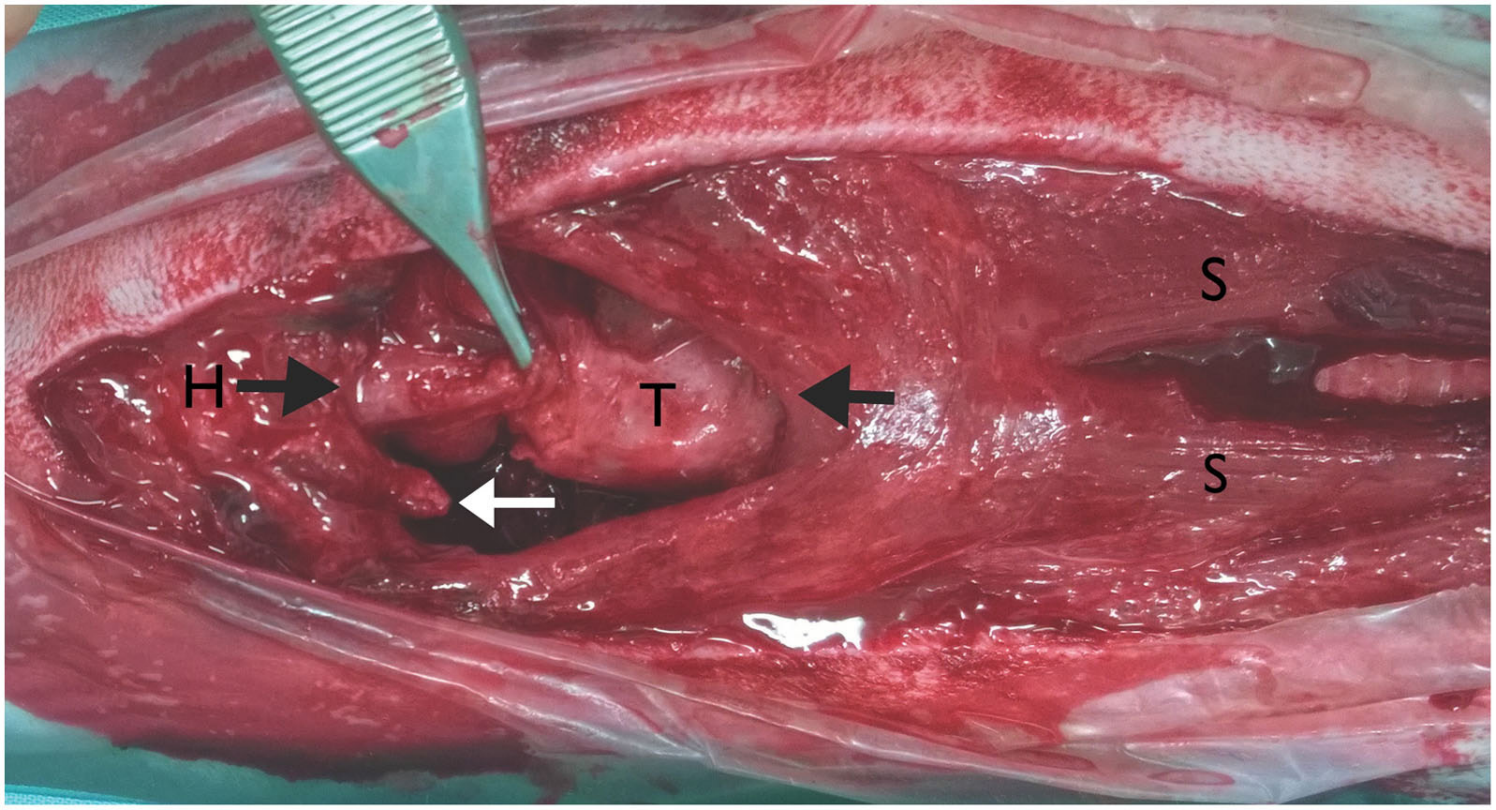
**Intraoperative pictures; ventral approach to the larynx: the head is on the left side of the picture**. Reattachment of the ventral epiglottic mucosa, fracture of the right thyrohyoid bone (white arrow), basihyoid bone (H), thyroid cartilage (T), and sternohyoid muscle (S), the black arrows show the ends of the muscle tissue that were sutured back together.

An incision was made into the skin along the ventral midline extending from the caudal part of the mandible to the level of the fifth cervical vertebrae. The subcutaneous tissue cranial to the larynx, the rostral surface of the epiglottis, and part of the thyroid cartilage were exposed as a result of a bilateral rupture of the sternohyoid muscles, a partial cranial rupture of the superficial sphincter colli muscle, and a partial bilateral rupture of the thyrohyoid muscles. Further inspection revealed that the hyoepiglotticus muscles were damaged bilaterally, and the basihyoid bone was displaced rostrally due to contraction of the hyoglossus and geniohyoideus muscles (Figure 2). Bilateral thyrohyoid bone fractures could be identified (Figures 3 and 4).

There was a degloving injury of the complete rostro-ventral glosso-epiglottic mucosa, including the bilateral hyoepiglotticus muscles allowing a direct view onto the epiglottic cartilage. The mucosa was torn from the apex of the epiglottis; the tear spanned a length of ~5 mm. The bilateral hyoepiglotticus muscles could not be clearly identified (Figure 3). The fibrous tissue between the caudal end of the epiglottis and the rostral part of the thyroid cartilage had a 4-mm tear. Two small fragments of thyrohyoid bone remained attached to each side of the thyroid cartilage.

After thorough investigation a copious lavage with Ringer’s solution was performed. The detached apex of the epiglottic mucosa was sutured into position with 4-0 poliglecaprone-25 sutures in a simple interrupted suture pattern. The epiglottic cartilage was reattached to the thyroid cartilage using 3-0 polydioxanone-25 sutures in a simple interrupted suture pattern. Small holes were drilled in each end of the thyrohyoid bone near the fracture lines with a 1-mm K-wire. Multifilament stainless steel (3-0, intraosseous wire) was used to repair the thyrohyoid fractures. Some muscle fibers were reattached to the rostro-ventral mucosa of the epiglottis, which were assumed to be hyoepiglotticus muscle, using a simple interrupted suture pattern with 4-0 poliglecaprone-25 sutures. Torn thyrohyoid and sternohyoid muscles were reattached with 2-0 polydioxanone in a simple interrupted suture pattern. An incision was made between the third and fourth tracheal rings and a 6-mm tracheostomy tube was inserted into the tracheal lumen and sutured to the skin. A Penrose drain was placed into the wound from the cranial to the caudal end, exiting the wound through an additional skin incision at the caudal end of the wound. A second copious lavage with Ringer’s solution was performed. The wound was closed routinely proximal to the tracheostomy tube, but the distal aspect was left open over a length of ~5 cm. An (4-mm) esophageal feeding tube was inserted into the esophagus through a separate skin incision on the left side lateral to the wound and secured to the skin with a Chinese finger trap suture (polyamide 2-0).

**Figure 5:**
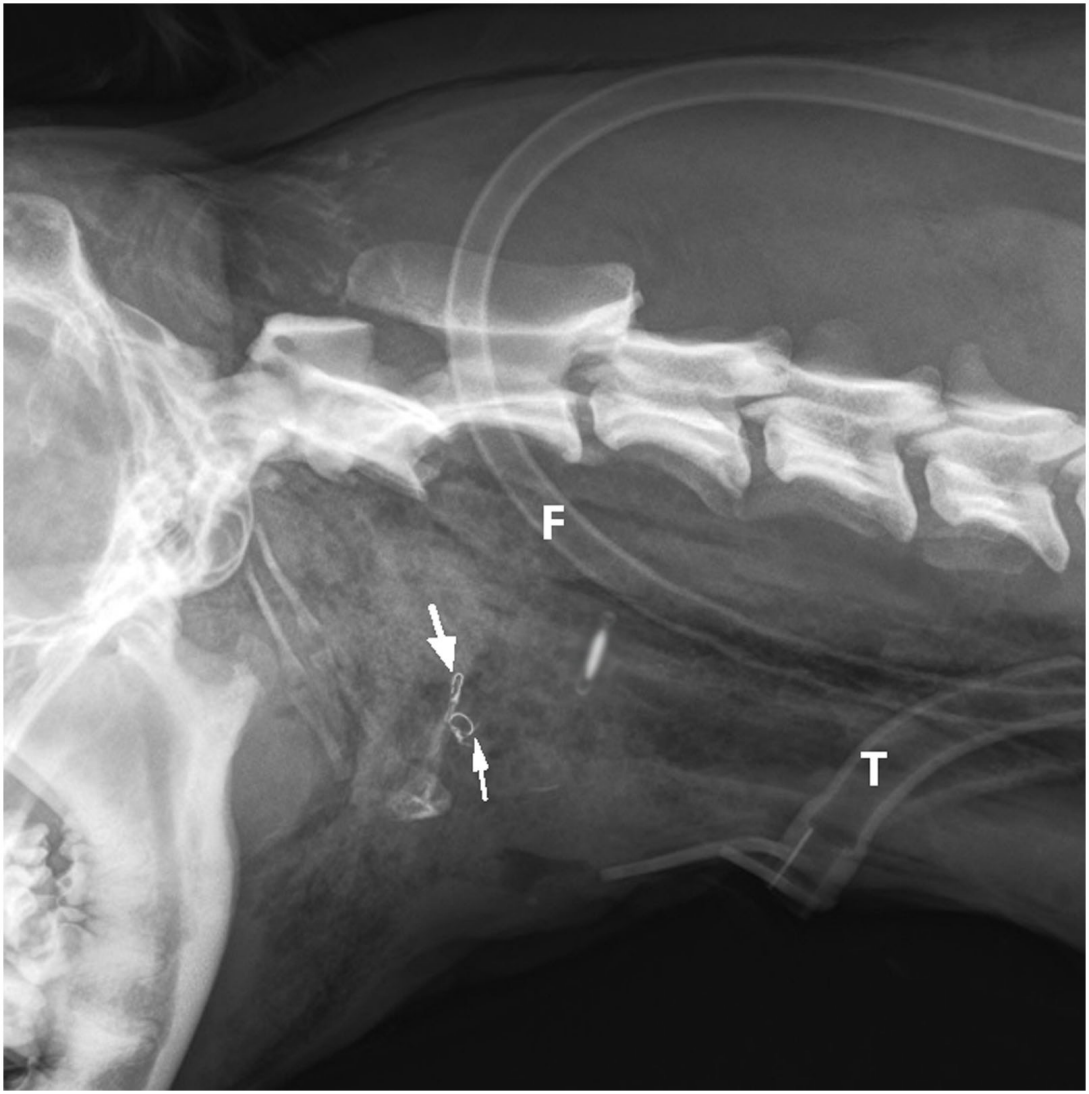
**Postoperative latero-lateral neck radiograph is showing the bilateral intraosseous wire fixation of the thyrohyoid bone fracture (arrows), feeding tube (F), and tracheostomy tube (T)**.

After surgery, latero-lateral cervical radiographs were taken (Figure 5) to confirm the position of the feeding tube in the esophagus, and a neck bandage was applied. The dog was taken to the intensive care unit for recovery and close monitoring. No further O_2_ supplementation was needed. The following medications were administered to the patient: buprenorphine (0.015 mg/kg IV, q 6 h) for the first 4 days, and metamizole (30 mg/kg IV, q 8 h) for the first 2 days; meloxicam (0.1 mg/kg SC. q 24 h) was added to the analgesia protocol on the second day and continued for another 5 days; amoxicillin/clavulanic acid (12.5 mg/kg IV, then orally, q 12 h) was administered for 10 days at a constant rate infusion with lactated Ringer’s solution (2 ml/kg/h) only for first 2 days to maintain homeostasis while the dog was unable to eat and drink.

For cleaning of the tracheostomy tube, 1 ml of 0.9% saline was injected into the tube every 2 h and immediately after injection, the fluid was aspirated with a surgical suction unit for 5 s. The dog was pre-oxygenated for 5 min before each cleaning procedure. The tracheostomy tube was exchanged twice daily during brief sedation with propofol (2–3 mg/kg IV) and additionally each time when the dog developed any breathing difficulties. A day after the surgery, esophageal tube feeding (150 kcal/day, mash feed 10 times a day) was started. On the second day, the amount was increased to 300 kcal/day, and from the third day, the dog was fed with 450 kcal/day.

The dog made a good recovery over the next few days postoperatively. On the second day, the dog was able to drink water. Seventy-two hours postoperatively, the tracheostomy tube and the Penrose drain were removed during brief sedation with propofol. Laryngeal function was assessed and sufficient bilateral abduction of arytenoid cartilage was noticed during inspiration. The dog was breathing very well without the tracheostomy tube; however, mild respiratory stridor was noticed. The remaining open wound (the caudal part of the surgical approach, including the wound of the tracheostomy tube) was managed subsequently with wet–dry bandages using polyhexanid fluid and gel until a healthy granulation tissue developed. Over the next few days, there was remarkable reduction in subcutaneous emphysema. On day 4, the dog regained his appetite and ate two meals a day of slurry tinned food.

On the fifth day, the dog was eating normally and his breathing had improved significantly, but an intermittent inspiratory stridor was still audible. Six days postoperatively, the feeding tube was removed. The wound of the caudal part of the surgical approach had a mild clear discharge and granulation tissue had developed. On the following day, the dog was discharged from the hospital, and it was recommended to continue with daily bandage changes with polyhexanid gel and non-adherent bandages at the primary vet. The secondary wound healing’s progress was additionally monitored at our hospital once a week.

**Figure 6:**
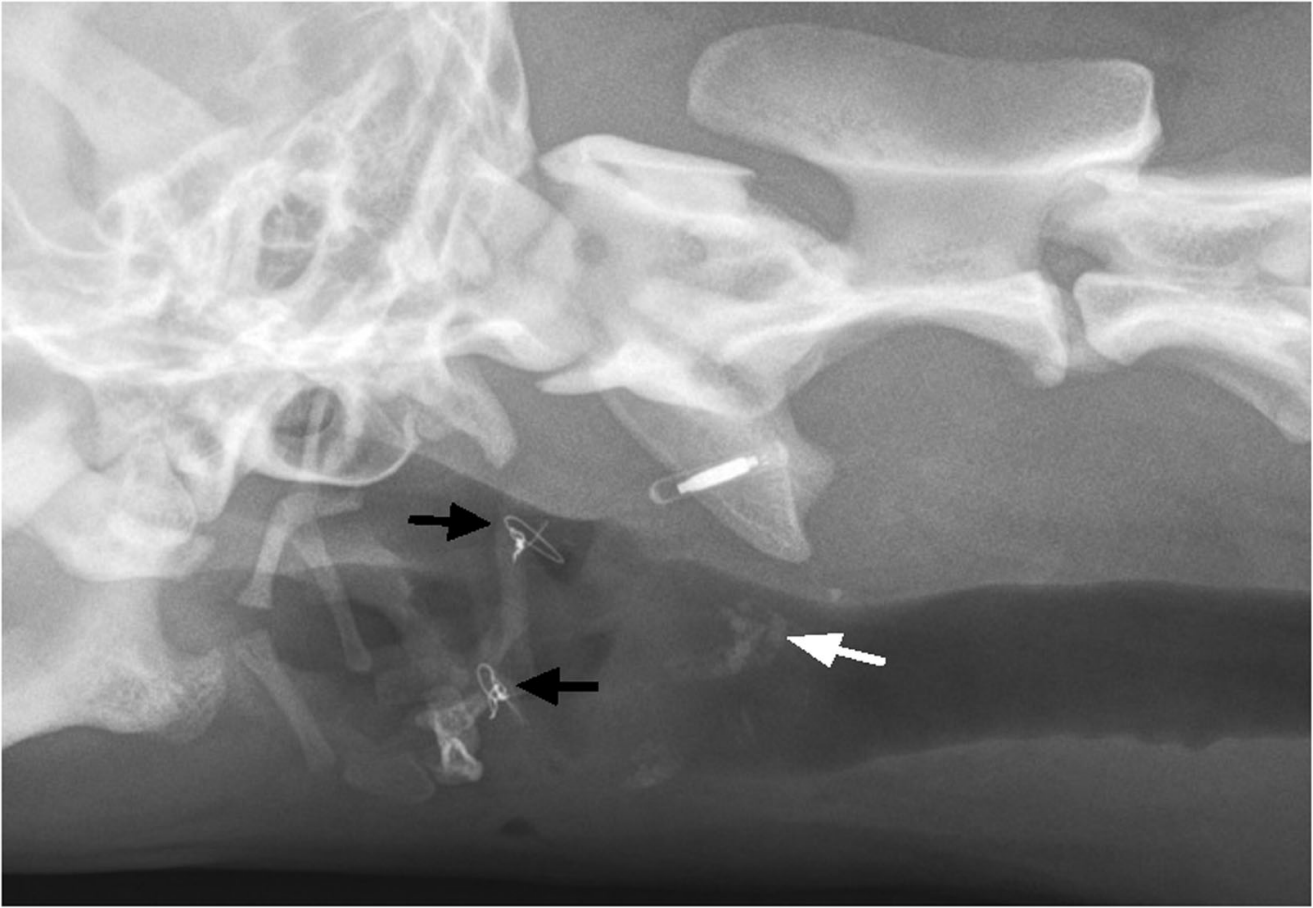
**Two-month postoperatively, oblique extended latero-lateral neck radiograph is showing bilateral thyrohyoid bone in presumptive normal anatomic position and intraosseous wire fixation (black arrows)**. Notice the ossification of laryngeal cartilage area (white arrow).

Two months after the surgery, the dog was re-examined. The owner reported that the dog exhibited normal swallowing, but was coughing occasionally at home. Also, palpation of the larynx was uncomfortable for the dog. Cervical radiographs revealed symmetry of the hyoid bones and laryngeal cartilage ossification (Figure 6). The owners declined sedation for repeated laryngeal examination of arytenoid cartilage function and further assessment of healing, such as a swallowing study with fluoroscopy or a computed tomography (CT) scan. They felt that their dog was clinically normal. At the 6-month follow-up, the owner reported normal breathing and swallowing. During the clinical examination, the dog showed no discomfort on palpation of the larynx.

## Background

Reports about laryngeal trauma are rare in the literature (1–5). Reported causes of laryngeal trauma in dogs include cervical bite injuries (1, 5, 6), road traffic accidents (3), and iatrogenic causes (2, 7, 8).

Clinical signs vary with the severity of laryngeal trauma; dyspnea and stridor have been documented in dogs (2, 3). Prior case reports of laryngeal bone problems in the veterinary literature are summarized in Table 1.

**Table 1:** **Summary of current literature about hyoid bone and laryngeal problems in dogs and cats**.

Localization	Publication	Number of patients	Signalment	Cause	Findings	Clinical signs	Treatment	Outcome, follow-up time
Hyoid bone problems	Milovancev and Wilson (7)	Five	Not specified	Ectopic thyroid carcinomas, surgical excision with partial hyoidectomy	Partial hyoidectomy, no specific details	Dysphagia resolved between 7 and 24 h after surgery	Ipsi-lateral ends of hyoid bone sutured with polypropylene	Clinically normal at long-term follow-up
	Lantz and Salisbury (8)	Three	Ø age of 7.3 years, German Shepherd dog, Australian Shepherd, and Border Collie	Ectopic thyroid carcinomas, surgical excision with partial hyoidectomy	Partial hyoidectomy, no specific details	Dyspnea in one dog. Dysphagia improved 6 days to 2 months after surgery in all dogs	No specific fixation of hyoid bone after partial hyoidectomy	Clinically normal at long-term follow-up
	Jordan et al. (5)	Two hyoid fracture cases in 55 dogs and cats with cervical bite wounds	Not specified	Bite injuries	Unknown type of hyoid bone fracture	Not specified	No details of surgical procedure	Not specified (53 of 55 animals survived to discharge)
	Levitt et al. (9)	One	3-year-old, male Shetland sheep dog	Not specified	Right stylohyoid disarticulation	Dysphagia and regurgitation	Surgical fixation (3-month post injury), 1 month later surgical excision due to recurrence of clinical signs	No dysphagia, euthanasia due to intermittent vomiting 4 months after resection
	Pass and Seltzer (6)	One	8-year-old, male miniature poodle	Bite injury	Bilateral hyoid bone fracture, no further details	Difficulty swallowing, mild dyspnea, and cutaneous puncture wounds	Conservative	Good, 3-month follow-up
	Manus (10)	Two	3- and 4-year-old, male German Shepherd dogs	Choke collars	Right epihyoid fracture	Enlarged tongue covered with blood, sublingual hematoma	Conservative	Good in case 1, 3-month follow-up Case 2 euthanized after 1 month
Laryngeal problems (cricoid, thyroid, and arytenoid)	Jordan et al. (5)	Two cases in 55 dogs and cats with cervical bite wounds	3-year-old, male neutered Jack Russell Terrier 6-year-old, Domestic Shorthaired cat	Bite injuries	Cricoid laceration Thyroid fracture	Subcutaneous and/or mediastinal emphysema	Cricoid laceration treated with fasciomuscular flap Primary repair	Discharged Discharged after revision surgery
	Monnet and Tobias (4)	One	Mixed breed dog	Bite injury	Unknown	Dyspnea, swelling, and laryngeal paralysis	Unilateral cricoarytenoid lateralization	Not specified
	Peppler et al. (1)	One of 45 dogs with cervical bite wounds	Not specified	Bite injuries	Cricoid fracture with laryngotracheal separation	Dyspnea	Primary repair	Euthanasia
	Doran and White (2)	One	3-year-old, female Golden Retriever	Iatrogenic after endotracheal intubation	Left arytenoid cartilage fracture	Episodes of inspiratory stridor and dyspnea	Unilateral cricoarytenoid and thyroaryteniod caudolateralization	Good, 7-month follow-up
	Francis et al. (3)	One	7-year-old, male neutered Labrador	Hit by car	Laryngeal collapse due to cricoid cartilage fracture	Changes in phonation and dyspnea	Conservative	Mild dyspnea when excited
Laryngeal problems (Epiglottis)	Jordan et al. (5)	One	4-year-old, Domestic Shorthaired cat	Bite injuries	Thyroepiglottic separation and epiglottic tear	Subcutaneous and/or mediastinal emphysema	Primary repair	Discharged
	Peppler et al. (1)	One dog with epiglottic trauma of 45 dogs with cervical bite wounds	Not specified	Bite injuries	Thyroepiglottic separation with tracheal and esophageal laceration	Dyspnea	Primary repair	Discharged after revision surgery
	Skerrett et al. (11)	24 dogs	Commonly middle-aged to older, small breed, and spayed females	Several hypotheses	Obstruction of the rima glottidis during inspiration caused by epiglottic retroversion	Stridor, dyspnea, coughing, and cyanosis (intermittent or constant)	– 18 dogs epiglottopexy – 1 dog subtotal epiglottectomy – 5 dogs medical management	Overall >2 years’ median survival time
	Mullins et al. (12)	One	6-year-old male neutered Yorkshire Terrier	Several hypotheses	Epiglottic retroversion	Dyspnea	After failure of epiglottopexy on two occasions, subtotal epiglottectomy performed	Normal eating and breathing, 7-month follow-up
	Flanders and Thompson (13)	Two	8-year-old castrated male Boxer 10-year-old spayed female Yorkshire Terrier	Several hypotheses	Epiglottic retroversion	Dyspnea	Epiglottopexy	Normal eating and breathing, 6 weeks’ follow-up Normal eating, occasionally stridor, 4-month follow-up
	De Lorenzi et al. (14)	One	13-year-old female mixed-breed dog	–	Epiglottis chondrosarcoma	No clinical signs, incidental finding	Total epiglottectomy	Clinically normal, 2-month follow-up

As a brief functional description, the hyoid bones hold the tongue and the larynx in position (15). During swallowing, the hyoid bones and the larynx move forward (rostrally) due to contraction of the bilateral geniohyoideus muscles and the hyoid apparatus. Simultaneously, the tip of the epiglottis is pulled caudally in order to close off the rima glottidis (Figure 2) (15).

## Discussion

This case report describes diagnosis and treatment of a dog with a cervical bite wound causing a bilateral thyrohyoid bone fracture and laryngeal trauma.

One study describing cervical bite wounds in dogs recommended surgical exploration through a ventral cervical midline approach in all cases with pneumomediastinum or subcutaneous emphysema (5). Moreover, surgical exploration is required if injury to deeper structures is suspected in these bite wounds (5). In our case, surgical exploration was chosen due to the subcutaneous emphysema with pneumomediastinum noticed on radiographs and the suspicion of epiglottic injury after endoscopic exploration.

The hyoepiglotticus muscle abducts the epiglottis from the rima glottidis (16). Laxity of this muscle may result in epiglottic retroversion and airway obstruction (4, 13). Recommended treatment for these cases is an epiglottopexy in the horizontal position (13). The hyoepiglotticus muscle was not clearly identifiable intraoperatively. The authors concluded that reattaching the mucosa to the rostral aspect of the epiglottis would result in an epiglottopexy, which has resulted in an acceptable clinical outcome in previous reported cases (11, 13). In two case reports, total or partial epiglottectomy were well tolerated (12, 14).

On 2-month follow-up radiographs, areas of ossification of the laryngeal cartilages became evident (Figure 6). The origin of cartilage ossification is difficult to elucidate. Ossification could have been caused by a cartilage fracture or an inflammatory process. In humans, post-traumatic ossification of laryngeal cartilages has been described (17).

Computed tomography may be more appropriate for elucidating laryngeal cartilage fractures. In human medicine, a CT scan is considered a technique that is more suitable than endoscopy for diagnosis and decision-making about surgical treatment of a laryngeal cartilage injury. Cricoid cartilage fracture has been diagnosed with a CT scan in one dog (3). Magnetic resonance imaging (MRI) is needed in some cases, such as epiglottic avulsion, particularly in cases where the cartilage cannot be clearly imaged with CT (17, 18).

In our case, the endoscopic and surgical exploration did not show any evidence of deeper injury to the laryngeal cartilage. However, a CT scan would have been preferable to assess the cartilage fully and might have detected any injuries which might have led to subsequent cartilage ossification. Therefore, in cases of laryngeal trauma, the authors recommend advanced imaging, such as CT.

The hyoid bone could be identified in its normal position during the 2-month follow-up, but it was difficult to assess the fracture line on the radiographs. An occasional cough of unknown origin and a mild reaction of discomfort on palpation of the laryngeal area were documented. The authors suspect that the coughing could be related to the surgical intervention at the epiglottis and its restricted movement. The mild pain on palpation of the larynx was assumed to be due to an incompletely healed laryngeal cartilage lesion, as there was radiographic evidence of post-traumatic cartilage ossification.

## Concluding Remarks

Laryngeal and hyoid bone injuries in dogs are rare. In the present case, reconstructive surgery resulted in an acceptable functional outcome.

## Author Contributions

GM contributed to writing the manuscript and literature review; additionally, he was the primary surgeon of the case. MB contributed to writing the manuscript. MS contributed to writing the manuscript. AA contributed to writing the manuscript as well as describing the anatomic structures. LB contributed to writing the manuscript as well as interpreting and describing the imaging findings. SS contributed to writing the manuscript and assisted with the surgery on the dog.

## Conflict of Interest Statement

The authors declare that the research was conducted in the absence of any commercial or financial relationships that could be construed as a potential conflict of interest. The reviewer SS and handling editor declared their shared affiliation, and the handling editor states that the process nevertheless met the standards of a fair and objective review.
